# Effect of integrating maternal health services and family planning services on postpartum family planning behavior in Ethiopia: results from a longitudinal survey

**DOI:** 10.1186/s12889-019-7703-3

**Published:** 2019-11-04

**Authors:** Linnea A. Zimmerman, Yuanyuan Yi, Mahari Yihdego, Solomon Abrha, Solomon Shiferaw, Assefa Seme, Saifuddin Ahmed

**Affiliations:** 1 Department of Population Family and Reproductive Health, Johns Hopkins Bloomberg School of Public Health, 615 N Wolfe St, Baltimore, MD USA; 2 Department of International Health, Johns Hopkins Bloomberg School of Public Health, 615 N Wolfe St, Baltimore, MD USA; 3 PMA2020-Ethiopia, Addis Ababa University, Addis Ababa, Ethiopia; 4 School of Public Health, Wolaita Sodo University, Wolaita, Ethiopia; 5 Department of Reproductive Health and Health Service Management, School of Public Health, Addis Ababa University, Addis Ababa, Ethiopia

**Keywords:** Postpartum family planning, Integration, Maternal health, Ethiopia, Postnatal care, Contraceptive use, Longitudinal data

## Abstract

**Background:**

Very few postpartum women want to become pregnant within the next 2 years, but approximately 60% of postpartum women in low- and middle-income countries are not using contraceptive methods. The World Health Organization recommends that women receive postpartum family planning (PPFP) counseling during antenatal, immediate postpartum, and postnatal services. Our objective was to establish whether PPFP counseling is being provided in antenatal and postnatal care services in SNNPR, Ethiopia and whether receipt of PPFP counseling improved uptake of postpartum family planning use by 6 months postpartum.

**Methods:**

Longitudinal data from the Performance Monitoring for Accountability 2020 – Maternal and Newborn Health study were used. At screening, 329 women were identified as six or more months pregnant; 307 completed the survey at 6 months postpartum. We used weighted parametric survival analysis with Weibull distribution to assess the effect of receipt of postpartum counseling in antenatal and/or postnatal care by 6 weeks postpartum on contraceptive uptake, after adjusting for intention to use family planning, wantedness of the index pregnancy, delivery location, amenorrhea, exclusive breastfeeding, residence, parity, and education.

**Results:**

Coverage of PPFP counseling is low; by six-weeks postpartum only 20% of women had received counseling. Women who received counseling in postnatal care only and postnatal care and antenatal care took up contraception at significantly higher rates than women who did not receive any counseling (HR: 3.4, *p* < .01 and HR: 2.5, *p* = .01, respectively). There was no difference between women who received PPFP counseling only in ANC and women who did not receive counseling at all. Women who did not want the child at all took up contraception at significantly lower rates than women who wanted the child at that time (HR: 0.3, *p* = .04). Women who had four or more children took up contraception at significantly lower rates than woman with 1–3 children (HR: 0.3, *p* = .01). There were no significant differences by delivery location, exclusive breastfeeding, residence, or education.

**Conclusion:**

Integration of postpartum family planning counseling into postnatal care services is an effective means to increase postpartum contraceptive uptake, but significant gaps in coverage, particularly in the delivery and postnatal period, remain.

## Background

Pregnancies occurring within 24 months of a previous birth are associated with increased risk of serious maternal and newborn morbidity and mortality [1–7]. Family planning programs have increasingly recognized that the extended postpartum period, generally defined as the first year following birth, is a critical time to provide contraceptive methods to women in order to reduce unwanted pregnancies [2, 8, 9]. Demographic and Health Survey data (DHS) suggest that very few postpartum women want to become pregnant within the next 2 years (3–8%), but approximately 60% of postpartum women in low and middle income countries who want to delay childbearing are not using contraceptive methods [8, 10, 11]. To improve uptake of postpartum family planning (PPFP), the World Health Organization (WHO) recommends that women receive counseling during the antenatal, immediate postpartum, and the postnatal period, preferably integrated into a comprehensive maternal and newborn health (MNH) package [2].

Results of the impact of integration of PPFP services into maternal health services on uptake of contraceptive methods are mixed, however. Retrospective studies relying on DHS data have found a relationship between antenatal care (ANC) care and later contraceptive adoption, but whether or not PPFP counseling was actually provided during ANC services was not asked by the DHS [12, 13]; it is thus difficult to ascertain whether the relationship is due to common underlying positive factors that affect both behaviors or that the ANC exposure positively affects postpartum contraceptive adoption. Keogh and colleagues (2015) found that integration of PPFP counseling into ANC did not result in higher rates of contraceptive use in the postpartum period, but did find a significant impact on increasing women’s intention to use contraceptives [14]. In their 2015 review, Cleland and colleagues note that single, short counseling sessions do not appear to improve PPFP uptake, but repeat, “high-intensity” antenatal care counseling might [15]. Integration into delivery care and postnatal care has shown more consistently positive impacts on increasing postpartum contraceptive use; when PPFP counseling and provision is integrated into delivery and immediate postpartum services, there is evidence that a substantial proportion of women will choose to use contraception [10, 16–19]. Immediate postpartum provision of contraception remains a challenge, however. A lack of trained providers, provider bias, misconceptions about the safety of using contraception while breastfeeding, and competing demands on the healthworker have been shown to affect the coverage and quality of immediate postpartum family planning services [10, 20, 21] Receipt of postnatal services has also shown largely positive associations with postpartum contraceptive uptake [18, 22–24], but low provision of postnatal care (PNC) remains a persistent challenge [10].

Postpartum family planning uptake remains low in Ethiopia; in 2016, it was estimated that 21% of postpartum women were using family planning at 6 months postpartum and 26% of women at 1 year [25]. This number differed substantially by place of delivery; 39% of women who delivered in a health facility used contraception by 6 months postpartum, relative to 13% of women who delivered at home [25]. The DHS estimates, however, that only 33% of women in Ethiopia delivered in a health facility and an even lower percentage of postpartum women (19.1%) reported receiving postnatal care [26]. These estimates also do not account for confounding by wealth or residence; that is, women who are wealthier are both more likely to delivery in health centers and to use family planning methods, masking any causal association between accessing MNH services use and PPFP uptake.

To address the low use of the formal health system in rural areas, Ethiopia introduced an ambitious community health program, relying on Health Extension Workers (HEWS) to provide a range of community- and facility-based services, including family planning counseling and provision. HEW activities include the provision of range of services, including family health, hygiene and sanitation, and reproductive and maternal health, including ANC and PNC. A time use study by Mangham-Jefferies and colleagues found that on average, HEWs spent approximately one-quarter of their time on family planning and maternal, newborn, and child health activities, far exceeding other health areas [27]. Postpartum family planning counseling is included as a component of both ANC and PNC services offered by HEWs and is intended to be integrated into all levels of facility based maternal care in Ethiopia [28]. Guidance documents name postpartum family planning counseling as components to be included in the third and fourth antenatal care visits, ideally occurring during the second trimester [29]. Despite the emphasis on integration, most large, population-based surveys, such as DHS and MICS, do not ask whether ANC and PNC included PPFP counseling, the coverage of PPFP counseling is not well known. To fill the knowledge gap of the coverage of specific components of care, including PPFP counseling, the Performance Monitoring and Accountability Maternal and Newborn Health (PMA-MNH) study was conducted in Southern Nations Nationalities and Peoples Region (SNNPR) in July 2016. It employed a longitudinal design, following pregnant women from the 6 month of pregnancy through 6 months postpartum, and is ideal to assess whether and when PPFP counseling was provided and its association with postpartum contraceptive uptake.

Our objective in this paper is to examine whether PPFP counseling is being provided during antenatal and postnatal care services and whether receipt of PPFP counseling in these services improved uptake of postpartum family planning use by 6 months postpartum.

## Methods

### Data

The PMA-MNH study was conducted in Southern Nations Nationalities and Peoples Region (SNNP-R) between July 2016 and July 2017. It expanded on the previously implemented Performance Monitoring and Accountability 2020 (PMA2020) survey, a cross-sectional survey fielded in Ethiopia since 2013, by implementing a longitudinal component [30]. Forty-four enumeration areas (EAs) used in PMA2020/Ethiopia were included in the sample. PMA2020 surveys used a multi-stage stratified cluster design wherein all EAs were randomly selected using probability proportional to size and all households were screened to identify any women six or more months pregnant. All consenting pregnant women were interviewed at screening, seven-days, six-weeks, and six-months postpartum by trained enumerators using smartphones programmed with Open Data Kit (ODK). At screening, 329 women from 10,399 households in 43 EAs were identified as eligible for the study and approached for consent; all women consented and enrolled. Over 6 months, eight women were lost to follow-up. Women who experienced a stillbirth or whose infants died during the study period (14 total) were excluded from analysis as their contraceptive behavior is likely to be different from women who did not experience an adverse event. In total, data from 307 women who completed the study were included in this analysis.

Household characteristics were assessed during enrollment. Questions regarding intention to use contraception, previous use of contraception, and intended delivery location were also assessed at enrollment. Components of antenatal care, postnatal care by 7 days, and delivery care were assessed at the first follow-up visit (< 7 days). Uptake of postpartum family planning, including method choice, was assessed at 6 weeks and 6 months postpartum, in conjunction with receipt of postnatal care, amenorrhea status, and if relevant, dates of contraceptive initiation, return of menses, and sexual resumption.

### Analyses

The outcome of interest was time to uptake of a modern method of contraception, within 6 months postpartum. Modern contraceptive use was defined as use of male/female sterilization, IUD, implants, pills, injectables, condoms or Lactational Amenorrhea Method (LAM). Two of four women who reported using LAM were reclassified as non-users as they reported that their menstrual cycle had restarted and/or that they had not exclusively breastfed their infant in the previous 24 h. Women who reported using a modern method of contraception were asked when they started using the method. Parity was classified as either first birth, 2–3 births, or 4 or more births. At the seven-day, six-week, and six-month interview, women reported separately on postnatal care received for their infant and postnatal care received for themselves. For the purposes of this analysis, these were combined to indicate whether a woman had received postnatal care for any reason by the six-week visit and whether postpartum family planning counseling was delivered as part of that visit.

Survey weights were used to account for complex survey design and generate regionally representative estimates of postpartum women, including for the modern contraceptive prevalence rate and distribution of respondent characteristics. We used weighted parametric survival regression analysis with Weibull distribution to assess the hazards of modern contraceptive use and associated covariates of interest. The primary independent variable was receipt of PPFP counseling in MNH services, defined as whether the woman received no PPFP counseling, received counseling in ANC only, received counseling by 6-weeks postpartum PNC only, or received counseling in both ANC and PNC. Due to small sample sizes, we did not differentiate women who did not receive any ANC and PNC services from women who did receive services but did not receive PPFP counseling. Sensitivity analyses were conducted to assess whether there were significant differences between these groups by running the models (described below) with and without differentiating these groups. Additional independent variables include the respondent’s intention to use family planning in the future (obtained at enrollment), whether the index pregnancy was wanted then, later, or not at all (obtained at enrollment), delivery location (facility or home), amenorrhea at 6 months, exclusive breastfeeding at 6 months, parity, education, and residence. No data for the variables of interest were missing. Though included in bivariate analyses, age and wealth were not included in the final model due to significant correlation with parity and urban/rural residence, respectively. Parametric survival models with a Weibull distribution were used to adjust for multiple variables.

## Results

Table 1 shows the background characteristics of women who enrolled in the study and the prevalence of amenorrhea, sexual activity, exclusive breastfeeding, and contraceptive use at 6 months postpartum. There were no significant differences in sociodemographic characteristics, gathered at baseline, between women who completed the final interview at 6 months and those who were lost to follow-up or dropped for adverse events and thus only women who completed all three interviews are included in the table below. Half of the women included in the panel were between age 25–34 and almost all were married. The sample was predominantly rural and over half of women had four or more births, including the index birth. The majority of women either did not attend school (44.4%) or attended only primary school (45.3%).

**Table 1:** Background characteristics of women in analytic sample

	Weighted n	Weighted %
Total	307	100
Age group		
15–24	100	32.4
25–34	160	52.1
35–49	47	15.5
Married	300	97.8
Urban	34	11.2
Parity^a^		
1	63	20.7
2–3	79	25.7
4 or more	165	53.6
Educational Attainment^a^		
Never attended	136	44.4
Primary	139	45.3
Secondary or Higher	32	10.3
Pregnancy wantedness		
Then	181	59.0
Later	96	31.4
Not at all	29	9.6
Intend to use FP in the future	263	85.6
Six months postpartum		
Amenorrheic	244	79.4
Sexually active	270	87.9
Exclusive breastfeeding (24 h)	50	16.3
MCPR	128	41.5

^a^ Data collected at 7-day postpartum interview

At enrollment, approximately 40% of women reported that they either wanted the pregnancy at a later time or not at all and 85% of women reported that they intended to use a contraceptive method at some point in the future. By 6 months postpartum, 87.9% of women were sexually active and only 16.3% reported exclusively breastfeeding their child in the 24 h before the survey. Approximately 80% were amenorrheic and 41.5% reported that they were currently using a modern method of contraception.

Table 2 shows the health service utilization and receipt of PPFP counseling from antenatal care through 6 months postpartum. Receipt of antenatal care was significantly higher than receipt of postnatal care coverage (83.6% versus 8.5, 30.3 and 63.5% by 7 days, 6 weeks, and 6 months, respectively). The number of women who reported receiving 4 or more ANC visits (58.7%) was markedly less than those who reported receiving any ANC. About half of women who received antenatal care reported receiving PPFP counseling; this was similar for women who reported receiving postnatal care at 7 days and postnatal care at 6 weeks. By 6 weeks postpartum, 43.0% of women had not received postpartum family planning counseling in either ANC or PNC, 34.7% received it in ANC only, 9.8% received it in PNC only, and 12.5% of women had received PPFP counseling in both visits.

**Table 2:** Health service utilization and postpartum family planning counseling by interview

	Percent
Seven day follow-up	100.0
Any ANC	83.6
4+ ANC	58.7
PPFP counseling (among women receiving ANC)	56.5
Facility delivery	52.1
PNC within 7 days	8.5
PPFP counseling (among facility delivery or women receiving PNC)	56.5
Six week follow-up	
Any PNC	30.3
PPFP counseling in PNC	50.2
PPFP counseling at six-weeks	
No ANC or PNC counseling	43.0
ANC counseling only	34.7
PNC counseling only	9.8
ANC and PNC counseling	12.5
Six-months post-partum	
Any PNC	63.5
PPFP counseling in PNC	64.8

**Fig. 1:**
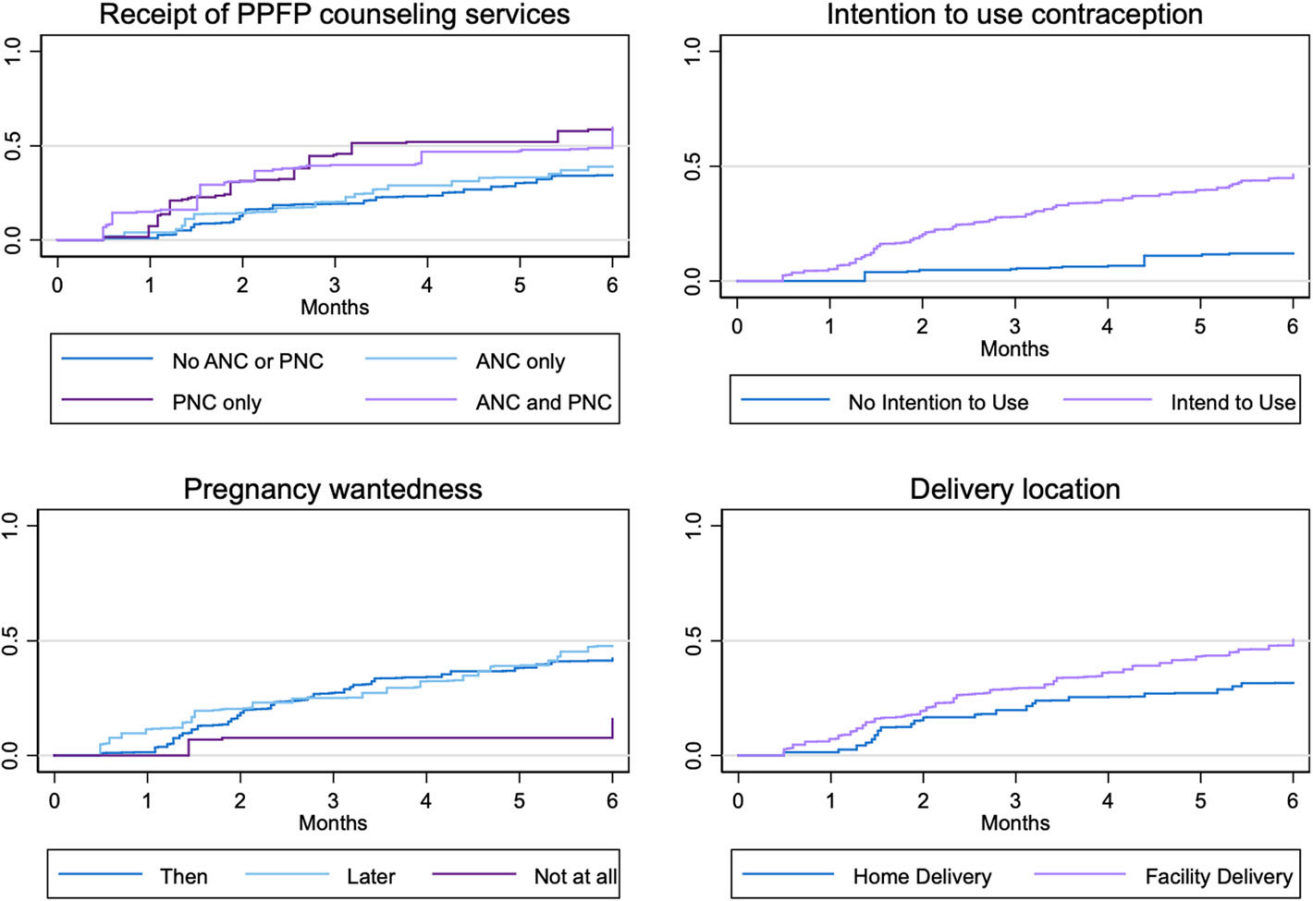
Kaplan-Meier failure function by receipt of PPFP counseling, contraceptive intention, pregnancy wantedness, and delivery location

Figure 1 below shows the Kaplan-Meier failure function for uptake of a contraceptive method over the six-month period, by receipt of PPFP counseling, intention to use contraception, wantedness of the index pregnancy, and delivery location. Women who received no PPFP counseling or who received counseling only in ANC had similar rates of contraceptive uptake over the six-month period, with fewer than half of women using contraception by the end of the 6 months, while women who received PPFP counseling in PNC only or ANC and PNC had faster rates of uptake. As shown in Table 3, the hazard (instantaneous risk rate) of contraceptive uptake was statistically significantly higher among women who received counseling in PNC alone relative to women who did not receive any PPFP counseling (HR: 2.3, *p* = .03). The hazard was also significantly higher among women who reported intention to use contraception at enrollment relative to women without that intention (HR: 5.0, *p* < .01). Women who reported that they did not want the index pregnancy at all had lower hazards of uptake compared to women who reported that they did want to become pregnant at that time, but the difference is not statistically significant (HR: 0.3, *p* = .10). Women who delivered in a facility had a 1.8 times higher hazard than women who delivered at home, but the difference is only marginally significant (*p* = .08).

**Table 3:** Hazard ratios for time to contraceptive uptake within 6 months postpartum, bivariate and adjusted

	Bivariate		Adjusted	
	HR	p-value	HR	p-value
PPFP counseling (ref: No ANC or PNC counseling)				
ANC counseling only	1.2	0.75	1.3	0.37
PNC counseling only	2.3	0.03	3.4	<.01
ANC and PNC counseling	2.2	0.07	2.5	0.01
Intend to use family planning in the future	5.0	<.01	4.7	0.01
Pregnancy wantedness (ref: Then)				
Later	1.1	0.56	1.1	0.82
Not at all	0.3	0.10	0.3	0.04
Facility delivery	1.8	0.08	1.3	0.36
Return of menstrual cycle	4.5	< 0.01	2.9	<.01
Exclusive breastfeeding	0.7	0.33	1.1	0.77
Urban (ref: rural)	2.3	<.001	0.8	0.42
Age (ref: 15–24)				
25–34	0.6	0.09		
35–49	0.3	<.01		
Parity (ref: 1)				
2–3	1.0	0.90	1.0	0.89
4 or more	0.3	<.01	0.3	<.01
Education (ref: Never)				
Primary	2.2	0.01	1.0	0.96
Secondary or higher	3.5	<.01	1.3	0.56
Wealth (ref: Poorest)				
Wealthier (ref: Poorest)	2.2	<.01		
Wealthiest	3.5	<.01		

In terms of sociodemographic characteristics, younger, urban, more educated, lower parity, and wealthier women were more likely to adopt a contraceptive method than older, rural, less educated, high parity women, and poor women, respectively.

After adjustment for background characteristics (shown in Table 3), the relationship between receipt of PPFP counseling and uptake strengthened; women who received counseling in PNC only and PNC and ANC took up contraception at significantly higher rates than women who did not receive any counseling (HR: 3.4, *p* < .01 and HR: 2.5, *p* = .01, respectively). The effect of intending to use family planning in the future also remained consistent. The association between pregnancy wantedness and contraceptive uptake was strengthened after adjustment; women who did not want the child at all took up contraception at significantly lower rates than women who wanted the child at that time (HR: 0.3, *p* = .04). Women who reported that their menstrual cycle returned had 2.9 times the hazard of contraceptive uptake than women who were amenorrheic at 6 months (*p* < .01). High parity remained statistically significant; women who had four or more children took up contraception at significantly lower rates than woman with 1–3 children (HR: 0.3, *p* = .01). There were no significant differences in the hazard of contraceptive uptake by delivery location, exclusive breastfeeding, residence, or education.

## Discussion

This study examined both the coverage and effect of receiving PPFP counseling into maternal and newborn health services in SNNP region, Ethiopia. Overall, we found that receipt of postpartum family planning counseling in postnatal care services is an effective means to increase postpartum contraceptive uptake, but that significant gaps in coverage, particularly in the delivery and postnatal period, remain a challenge. We found no effect of receiving postpartum family planning counseling in antenatal care or on delivering in a health facility on contraceptive uptake. Of note, women who did not want the index pregnancy at all and women who had four or more children had the lowest rate of modern contraceptive uptake by 6 months postpartum.

Postpartum family planning use is relatively low in SNNP region; fewer than 45% of women were using a method by 6 months postpartum, despite approximately 90% of women having resumed sexual activity. In general, we found higher use of maternal and newborn health services than reported elsewhere for SNNP. The majority of women (83.5%) reported that they received at least one antenatal care visit in SNNP and delivered in a facility (52.1%), higher than the DHS 2016 regional estimates of 69.3 and 33.2% of women, respectively [26]. These numbers are not entirely comparable, however; PMA Ethiopia data were collected within 7 days of birth and only included births taking place between July and September 2017, while the DHS included all births to women in the 3 years prior to the survey. While the DHS thus has a larger sample size, recent trends towards higher uptake of health services may be masked by the inclusion of births occurring 3 years before.

Though the numbers of women receiving maternal and newborn health services may be increasing, the coverage of PPFP counseling remains low. Half of women who received ANC and/or PNC services reported receiving any PPFP counseling during these visits, indicating a significant missed opportunity for counseling. Our results indicate that it is particularly important to improve both the coverage and content of PNC services. Women who received counseling on PPFP by 6 weeks postpartum took up modern contraception at significantly higher rates than women who did not, but by 6 weeks postpartum, only one in five women had received this counseling. These results are similar to those from other regions in Ethiopia that found both low rates of postnatal care coverage and a significant positive association of contraceptive use and postnatal care amongst postpartum women [22, 23, 31]. These results reinforce the importance of including PPFP counseling and provision into standard postnatal care services.

We found no association between receiving PPFP counseling in ANC services alone and PPFP uptake; this differs from studies that rely on DHS data [12, 13, 32], but is largely consistent with findings from smaller studies in Ethiopia and East Africa [14, 22, 23]. This lack of effect should not, however, be interpreted that introduction of PPFP counseling in ANC services is not worthwhile. When women reported at enrollment that they intended to use contraception in the future, they had significantly higher rates of uptake by 6 months postpartum. ANC serves as a valuable opportunity to introduce contraceptive methods and, as Keogh and colleagues demonstrated in Tanzania, strengthen the intention to use contraception in the future [14]. Evidence suggests that women who receive counseling multiple times in ANC may have higher rates of uptake than those who receive single sessions, underscoring the utility of providing multiple opportunities to provide information and strengthen intentions [15]. Though we could not differentiate between the number of times PPFP counseling was delivered, almost half of women received three or fewer ANC visits and, as current guidance is to counsel on PPFP at the third and fourth visit, it is likely that many women received information on PPFP counseling at only one visit. If PPFP counseling were to be introduced earlier into the ANC schedule, including as early as the first visit, the repeat exposure to counseling messages may encourage greater uptake, even among women with fewer than 4 ANC visits.

Consistent with the literature, we found that return of the menstrual cycle was one of the factors most strongly associated with contraceptive uptake [14, 33–35]. Evidence suggests that women wait to use contraception until their cycle has returned and are unaware that they are at risk of conceiving even if they are amenorrheic [11, 36]. PPFP counseling integrated into ANC provides an ideal opportunity to counsel on return to fertility, correct use of LAM, and pregnancy risk during the postpartum period while PNC is an opportunity to reinforce these messages. That return of the menstrual cycle remains so strongly related to use in this study demonstrates that women remain unaware of their ability to become pregnant before their cycle returns and highlights a potential gap in current PPFP programming.

We also found no effect of delivering in a health facility on PPFP uptake after adjusting for receipt of counseling in ANC and PNC care. This differs from some previous research using DHS data that found a significant association between institutional delivery and PPFP uptake at the national level [17, 32]. Immediate postpartum contraceptive counseling and provision did not appear to be widespread in SNNP; only 7% of women who delivered in a facility reported that they had spoken to anyone about family planning by the seven-day postpartum interview [not shown]. Given the evidence that postpartum services can be successfully integrated into delivery services [20, 37], this represents a significant missed opportunity.

The relationship of parity and contraceptive use is in keeping with cross-sectional PMA2020 data from 2017; married women with 4+ births had lower modern contraceptive prevalence rates (27.1%) than women who had 0–1 children (41.6%) or 2–3 children (43.6%) [38]. Prevention of unwanted pregnancies and reduction of high parity births, a priority to address due to increased risk of maternal and child mortality, can be reduced through improved contraceptive use practices, including postpartum family planning. Though sample size must be considered when interpreting results, high parity women and women who want to limit births in SNNP-R do not seem to be receiving effective counseling and services.

Our study is not without limitations, primarily due to the small sample size. To achieve adequate statistical power for analyses, we did not differentiate between women who received no services during the antenatal and postnatal period and those who did receive services but did not receive counseling. Sensitivity analyses of preliminary models demonstrated that the overall findings are consistent when disaggregated by no services versus no counseling, but model convergence was an issue in the final model. Additionally, the study was conducted only in one region of Ethiopia. Given the diversity between regions and groups within Ethiopia, these findings may not be generalizable to the country as a whole. Finally, while our study reduced bias related to selective retrospective recall, it cannot fully address selective receipt of PPFP services due to endogeneity. Endogeneity may occur, for example, in selective counseling of PPFP services due to a health worker’s knowledge of client’s motivation to use FP [39]. Our study, however, has a number of strengths, primarily the longitudinal design with low loss to follow-up. This allowed us to assess exposure to postpartum counseling and uptake of contraception at frequent intervals throughout the postpartum period, limiting recall bias. As we collected information on pregnancies occurring only between July 2017 and September 2017, we also limited confounding due to temporal changes that are present when including all births over a three- or five- year period, which may mask more recent progress. Finally, though the study may not be generalizable to all of Ethiopia, it was designed to be representative of currently pregnant women within SNNP and findings are generalizable within the region.

## Conclusion

Our results demonstrate that receipt of PPFP counseling during postnatal care visits is associated with higher rates of contraceptive uptake among recently postpartum women, an important programmatic finding. The success of integration is limited, however, by low repeat ANC attendance, low-uptake of postnatal care services and inconsistent provision of PPFP counseling. Additionally, the lack of effect seen for facility delivery indicates that immediate postpartum services are not yet widely utilized in Ethiopia. A significant gap in services remains among high parity women and women who reported that the birth was unwanted. Short birth intervals, high parity and unwanted births are still high in Ethiopia, and interactions with health providers during MNH care provide unique opportunity to improve low contraceptive use situation in the country. Postnatal care providers must be trained to provide comprehensive PPFP counseling, including immediately after delivery and during child health visits, such as well-baby and immunization visits, for all women and particularly those who are interested in limiting future fertility. Future research should identify barriers to accessing postnatal care and what counseling messages and services can most effectively reach high parity women. Improving the coverage and quality of postpartum family planning services can help to reduce the number of high-risk pregnancies, further contributing to global declines in unintended pregnancies and maternal morbidity and mortality.

## Data Availability

The datasets generated during the study are publicly available from the PMA website (https://www.pma2020.org/request-access-to-datasets).

## Abbreviations

ANC: Antenatal care
PMA: Performance monitoring and accountability
PNC: Postnatal care
PPFP: Postpartum family planning counseling
SNNP-R: Southern Nations Nationalities and People’s Region
